# Serum Anti-Müllerian Hormone: A Potential Semen Quality Biomarker in Stud Dogs?

**DOI:** 10.3390/ani12030323

**Published:** 2022-01-28

**Authors:** Guillaume Domain, Justyna Buczkowska, Patrycja Kalak, Eline Wydooghe, Penelope Banchi, Osvaldo Bogado Pascottini, Wojciech Niżański, Ann Van Soom

**Affiliations:** 1 Department of Internal Medicine, Reproduction and Population Medicine, Faculty of Veterinary Medicine, Ghent University, 9820 Merelbeke, Belgium; eline.wydooghe@ugent.be (E.W.); penelope.banchi@ugent.be (P.B.); osvaldo.bogadopascottini@uantwerpen.be (O.B.P.); ann.vansoom@ugent.be (A.V.S.); 2 Department of Reproduction and Clinic of Farm Animals, University of Environmental Science, Grundwaldzki Square 49, 50-357 Wroclaw, Poland; justyna.buczkowska@upwr.edu.pl (J.B.); 102726@student.upwr.edu.pl (P.K.); wojciech.nizanski@upwr.edu.pl (W.N.); 3 Veterinary Physiology and Biochemistry, Department of Veterinary Sciences, University of Antwerp, 2610 Wilrijk, Belgium

**Keywords:** dog, AMH, biomarker, semen quality

## Abstract

**Simple Summary:**

Assessing semen quality in dogs requires experience and specialized equipment. Therefore, this study investigates the potential value of measuring the blood concentration of Anti-Müllerian hormone (AMH), a hormone produced by Sertoli cells, to predict semen quality in dogs. Forty-five healthy dogs were included in this study and their age as well as different semen parameters were correlated to blood AMH concentration. Moderate negative associations were found between AMH and semen motility and morphology indicating that high serum AMH may be a potential biomarker for identifying which dogs would require further semen investigation. Future research is however needed to confirm these preliminary results.

**Abstract:**

Anti-Müllerian hormone (AMH) has been suggested to be involved in spermatogenesis. The aim of this study was to investigate the relationship between blood serum AMH concentration and semen quality in dogs. Moreover, this study sought to find the optimal cut-off point value of serum AMH with the greatest sensitivity and specificity to predict semen quality. Forty-five clinically healthy dogs were included in the study and their age as well as the following semen parameters were determined and correlated to serum AMH concentration: total sperm output, normal morphology, plasma membrane integrity, total motility, progressive motility, and velocity parameters. Statistical analysis for correlations were performed using Spearman’s correlation coefficients. Moderate negative associations were found between serum AMH and semen total motility (r = −0.38, *p* = 0.01), progressive motility (r = −0.36, *p* = 0.01), and normal morphology (r = −0.36, *p*= 0.02). Based on these associations, an AMH concentration of 5.54 µg/L was found to be the optimal cut-off point value to obtain the greatest summation of sensitivity (86%) and specificity (63%) to predict semen quality. The serum AMH assay may therefore be a potential hormonal marker to predict which dogs would require further semen analysis. Future research is however needed to confirm these preliminary results.

## 1. Introduction

Dog fertility is essential to avoid unsatisfactory breeding performance. In order to assess the potential fertility of a stud dog, a complete breeding soundness evaluation can be carried out. This evaluation consists of a detailed history, a thorough physical examination, ultrasonography of the genital tract, and semen evaluation [1,2]. Furthermore, hormonal testing can provide additional information in some cases [3]. However, semen collection is not always successful and semen analysis requires experience and specialized equipment, which is not available in all veterinary practices. For this reason, additional diagnostic tools such as the presence of particular biomarkers in a blood sample would be of interest for a general veterinary practitioner to evaluate gonadal function and to predict fertility in male dogs [4,5,6].

Anti-Müllerian hormone (AMH), also called Müllerian-inhibiting substance, is a glycoprotein of the transforming growth factor-β family secreted by Sertoli cells in males [7]. This family of proteins plays an important role in the regulation of cell proliferation and apoptosis in many different biological processes, including spermatogenesis [7,8,9]. During embryonic sex differentiation, immature Sertoli cells secrete a high concentration of AMH which induces the regression of Müllerian ducts, which otherwise would develop into female internal sex organs [9,10,11,12,13,14]. The immature population of Sertoli cells will then be responsible for the basal synthesis of AMH, which gradually decreases in concentration upon the onset of puberty [15,16,17]. At puberty, Sertoli cells acquire androgen receptors and AMH secretion decreases as a result of testosterone production, as demonstrated in humans [18], stallions [19], and bulls [20]. Besides testosterone, meiotic germinal cells have also been found to inhibit AMH secretion [21]. It is well-known that Sertoli cells exert an important role in fertility by providing protective and nutritive support to the meiotic germ cells. The assessment of AMH concentration has therefore been suggested to serve as a direct biomarker of Sertoli cell function and indirect biomarker of spermatogenesis [22,23,24,25,26,27].

The interest in AMH in the context of canine reproduction has significantly increased in recent years. In male dogs, serum AMH may be used to differentiate intact and cryptorchid dogs from castrated dogs. Intact dogs have a higher serum AMH concentration than castrated dogs but a lower serum AMH concentration than cryptorchid dogs [5,14,28,29]. The increased serum AMH concentration in cryptorchid dogs may be explained by the high amount of immature Sertoli cells in the retained gonad(s) [14]. Serum AMH serves also as a potential biomarker in the diagnosis of Sertoli cell tumor as a result of the increased AMH production from neoplastic Sertoli cells [4,6,30,31]. However, to the best of the authors’ knowledge, no research has yet been reported on the use of AMH as a biomarker to predict semen quality in dogs. Therefore, the aim of this study was to investigate the relationship between serum AMH concentration and semen quality in dogs with the hypothesis that higher AMH concentration would be found in lower quality semen. The age of the dog was also considered to investigate its possible relation to serum AMH concentration. Finally, this study aimed to identify an ideal cut-off value for serum AMH concentration to determine which dogs would require further semen examination.

## 2. Materials and Methods

### 2.1. Animals

Sixty intact and privately owned male dogs took part in the present study between January and April 2021 at the teaching hospital of Ghent University. All dogs were at least 1 year old, clinically healthy, and had not been sick or given medication in the last 6 months. Exclusion criteria were abnormal clinical examination, blood admixture in the third fraction of the ejaculate (prostatic fluid), secretory or excretory azoospermia, and unsuccessful semen collection. During data collection, five dogs presented secretory azoospermia and ten had an unsuccessful semen collection. These dogs were therefore excluded from the study and 45 dogs from 21 breeds were finally included in the final dataset of the study.

### 2.2. Semen Quality Assessment

The sperm-rich fraction of the ejaculate of each dog was collected by digital manipulation into plastic vials as described by Linde-Forsberg [32]. After collection, the volume of the ejaculate was measured, and the semen was immediately placed in an incubator at 37 °C. Semen quality parameters were then assessed within 10 min by a single operator.

#### 2.2.1. Concentration

Semen concentration was measured using the Nucleocounter-SP100 (ChemoMetec, A/S, Allerød, Denmark), according to the manufacturer’s instructions [33]. Briefly, a 10 µL aliquot of semen was diluted with 1 mL lysis reagent S100 (ChemoMetec, A/S, Allerød, Denmark) and, after mixing, was loaded into a cassette containing propidium iodide. The cassette was then inserted into the fluorescence detector of the machine and the semen concentration of the sample was reported. Total sperm output (TSO) was then obtained by multiplying the reported concentration by semen volume.

#### 2.2.2. Motility and Velocity

Motility and velocity parameters were measured using the computer-assisted sperm analysis system ISAS^®^v1 (Proiser, Valencia, Spain) equipped with a heated stage set at 37 °C and a 10× negative phase-contrast objective. A video digital camera (Proiser 782C) was mounted on the microscope to capture images and transmit them to a computer. Thirty consecutive, digitized images were obtained at a frame rate of 60 fps. Tail detection was activated for ignoring non sperm particles and particle area was set between 12 and 80 µm^2^. The number of objects incorrectly identified as spermatozoa was minimized by using the playback function. A spermatozoon was considered immotile when presenting a VAP < 10 µm/s and spermatozoa which deviated <50% from a straight line were designated as progressive.

For each analysis, samples were diluted with physiological saline solution to a working concentration of 40 × 10^6^ cells/mL [34,35]. A 4-µL droplet was then loaded in a pre-warmed ISAS^®^D4C20 disposable counting chamber (Proiser, Valencia, Spain) and five fields were captured and analyzed. For each field, five kinematic parameters were retained and the average was taken for the analysis: total motility (TM, %), progressive motility (PM, %), average path velocity (VAP, µm/s), straight line velocity (VSL, µm/s), and curvilinear velocity (VCL, µm/s).

#### 2.2.3. Morphology and Plasma Membrane Integrity

Morphology and plasma membrane integrity of spermatozoa were assessed on eosin/nigrosin stained smears under bright-field microscopy at 1000× magnification (Olympus BX51TF, Tokyo, Japan). One hundred spermatozoa were evaluated for each parameter and classified according to their plasma membrane integrity (intact or damaged) and their morphology (normal, abnormal head, abnormal midpiece/tail, proximal protoplasmic droplet, and distal protoplasmic droplet) [36,37]. 

### 2.3. Hormone Analysis

After semen collection and analysis, blood samples were drawn from the cephalic vein, transferred into serum gel tubes with clot activator (Greiner Bio-one, Kremsmünster, Austria), and placed at 4 °C for 30 min to allow clot formation. Tubes were then centrifuged at 2000× *g* for 5 min and sera were immediately stored at −80 °C until analysis in an external specialized laboratory (Algemeen Medisch Laboratorium, Sonic Healthcare Benelux, Antwerp, Belgium). Serum AMH levels were quantified by electrochemiluminescence immunoassay (ECLIA) using the Elecsys AMH Plus immunoassay on the cobas e411 analyzer (Roche Diagnostics International Ltd., Rotkreuz, Switzerland). Results were determined via a calibration curve generated by 2-point calibration and a master curve provided by the manufacturer. The analyzer automatically provided the AMH concentration and controls were performed for each analysis. Samples with AMH concentrations above the measuring range (>23 mg/L) were diluted to obtain a definite value. The intra- and inter-assay precision were ≤1.3 and ≤4.1%, respectively. The limits of blank, quantitation, and detection were 0.007 ng/mL, 0.030 µg/L, and 0.010 µg/L, respectively. The method was standardized against the Beckman Coulter AMH Gen II ELISA assay.

### 2.4. Statistical Analyses

Statistical analyses were performed using R v4.1.2, 2021 (R Inc., Boston, MA, USA). The normality of the distributions was verified using histograms and Shapiro–Wilks tests. The Spearman’s correlation coefficients (ρ) were used to assess the relationship between serum AMH and semen parameters or age of the dog. The level of significance was set at *p* < 0.05 for all analyses. Correlations were considered as follows: less than 0.2 negligible association, 0.2 to 0.29 weak association, 0.3 to 0.39 moderate association, 0.4 to 0.69 strong association, and greater than 0.7 very strong association [38]. The optimal cut-off point value of serum AMH, representing the value associated with the greatest summation of sensitivity and specificity to predict semen quality, was obtained using the package cutpointr after dichotomization of the data [39]. Different threshold values for dichotomization have been tested to find out which one had the best association with serum AMH. The threshold values have been defined based on the principle that an ejaculate with a progressive motility of at least 70% and a normal morphology of at least 80% is of adequate quality [40,41]. Moreover, an ejaculate containing less than 60% of morphologically normal spermatozoa should be qualified of poor quality as it may reflect disturbances in testicular and/or epididymal function [40,41]. 

## 3. Results

The median age of the dogs included in the study was 3 years (inter-quartile range (IQR): 2–4.5 years, range: 1–13 years). Analyses of correlations between serum AMH concentration and investigated parameters are summarized in Table 1. Total motility (*p* = 0.01), progressive motility (*p* = 0.01), as well as the percentage of spermatozoa with normal morphology (*p* = 0.02) showed a negative correlation with serum AMH concentration. For the other semen parameters and the age of the dog, no significant associations with serum AMH concentration were found although a trend for a negative correlation regarding semen velocity parameters and a positive correlation regarding TSO can be observed (Table 1).

**Figure 1:**
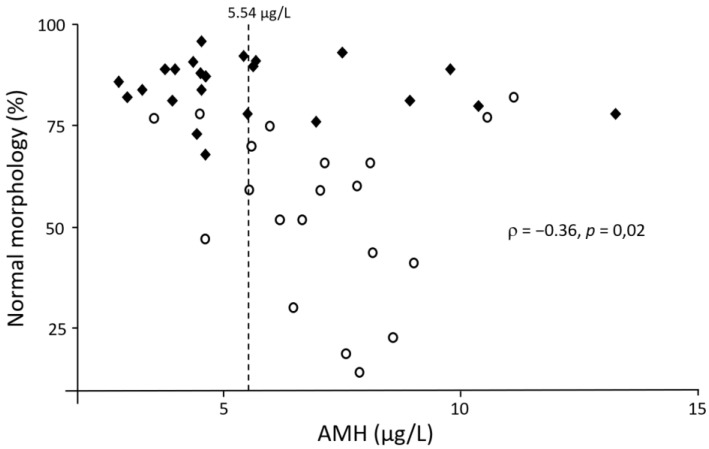
Scatter plot showing the relationship between serum AMH and the percentage of spermatozoa with a normal morphology. The dashed line represents the optimal cut-off value considering 60% normal morphology and 70% progressive motility as threshold values. Full diamonds represent dogs above these threshold values and empty circles represent dogs with at least one of the two parameters below these threshold values.

In view of the significant associations between serum AMH and some semen quality parameters, different threshold values were tested in this population to predict semen quality based on AMH concentration (Table 2). The inclusion of both morphology and progressive motility seemed to be the most effective to obtain the greatest summation of sensitivity and specificity. The prediction had a sensitivity of 86% and a specificity of 63% when 5.54 µg/L was taken as cut-off value to predict that a sample would have minimum 60% of morphologically normal spermatozoa and 70% of progressively motile spermatozoa. Furthermore, 83% of samples would at least satisfy these threshold values if serum AMH concentration is lower than 5.54 µg/L. Figure 1 represents the accuracy of these predictions.

## 4. Discussion

To the best of the authors’ knowledge, this is the first study to investigate the relationship between serum AMH and semen quality in dogs. We found that serum AMH concentration had a moderate negative association with semen quality. Total motility, progressive motility, and normal morphology were all negatively correlated with serum AMH concentration indicating that an increased AMH production may reflect abnormal Sertoli cell function and thus compromised spermatogenesis.

The association between serum AMH and fertility has already been well-documented in men but with conflicting results. A Danish study investigating 970 healthy young men observed that a significantly higher serum AMH was associated with lower percentage of morphologically normal spermatozoa [27]. However, this negative association between serum AMH concentration and semen quality was not reported consistently. Some studies found no differences [26,42,43,44,45,46], while others even reported a decrease in serum AMH in men suffering from subfertility and infertility in comparison to fertile men [47,48,49]. These conflicting results may be related to the reproductive disorder and the related Sertoli cell dysfunction encountered in men suffering from subfertility. A decrease in AMH concentration may results from a loss of functional Sertoli cells while an increased serum AMH concentration may result from a higher population of Sertoli cells that exhibit a prepubertal stage of development [42]. Such Sertoli cells with immature characteristics secrete AMH predominantly into the blood circulation through the basal layer instead of the seminiferous lumen [22,42]. Another explanation for an increased AMH may lie in the production of androgens given their role in the regulation of AMH production. Abnormal androgen production and/or absence or insensitivity of androgen receptors may impair AMH inhibition and explain the sustained high serum AMH production [17,50]. 

In the present study, higher serum AMH concentrations were associated with a lower percentage of morphologically normal spermatozoa, as previously described in men [27]. The absence of association between serum AMH and total semen output seems to be similar to men [26,27,43,44,45] but inter-species differences may be present as serum AMH also appeared to be negatively associated with semen motility, which has not been reported yet in men [27,45]. Interestingly, a positive correlation trend regarding TSO was found, which may reflect a compensation system to suboptimal semen quality in dogs. However, further research is required to confirm these results. 

In view of the significant associations between serum AMH concentration and some semen parameters found in this study, the cut-off value with the best summation of sensitivity and specificity was sought for different threshold values in order to evaluate the relevance of serum AMH as a (practical) biomarker of semen quality. The results found in the present study indicate that the best values were obtained when considering the semen to have a minimum of 60% morphologically normal and 70% progressive motile spermatozoa. Under these conditions, the best cut-off value to classify the quality of an ejaculate based on serum AMH was 5.54 µg/L. The latter was associated with an area under the curve of 0.71, which made the diagnostic accuracy of the test acceptable [51]. Thus, a serum AMH concentration below 5.54 µg/L would correctly predict that the semen would at least meet the threshold values for morphology and progressive motility in 83% of cases. The measurement of serum AMH concentration could therefore be a useful diagnostic tool to predict semen quality in dogs in places where semen cannot be collected or analyzed. However, when serum AMH concentration is higher than 5.54 µg/L, semen quality would only be below (at least) one of the threshold values in 67% of cases. For this reason, the authors would advise to perform a thorough semen evaluation when serum AMH concentration is higher than 5.54 µg/L.

## 5. Conclusions

The negative associations between serum AMH and semen motility and morphology found in this study make AMH a potential biomarker of spermatogenesis and thus predictor of semen quality in dogs when serum AMH concentration is low. However, further research is needed to confirm these preliminary results.

## Figures and Tables

**Table 1:** Spearman correlation coefficients (ρ) between serum AMH concentration and different semen parameters.

Parameter	Correlation (ρ)	*p*-Value
Total motility (TM)	−0.38	0.01
Progressive motility (PM)	−0.36	0.01
Curvilinear velocity (VCL)	−0.29	0.10
Straight line velocity (VSL)	−0.30	0.08
Average path velocity (VAP)	−0.32	0.06
Normal morphology (NM)	−0.36	0.02
Plasma membrane integrity	−0.06	0.70
Total sperm output (TSO)	0.28	0.06
Age of the dog	0.21	0.18

**Table 2:** Optimal cut-off point values with the greatest summation of sensitivity and specificity for different semen parameters.

Parameter	Cut-off AMH (µg/L)	AUC	Acc	Se	Sp	PPV	NPV
NM ≥ 80%	5.53	0.67	0.71	0.80	0.60	0.71	0.71
NM ≥ 60%	4.63	0.66	0.58	1.00	0.44	0.37	1.00
NM ≥ 80% + PM ≥ 70%	5.53	0.71	0.73	0.81	0.63	0.75	0.71
NM ≥ 60% + PM ≥ 70%	5.54	0.71	0.73	0.86	0.63	0.67	0.83

Assuming positive value are values above the cut-off value. Abbreviations: NM, normal morphology; PM, progressive motility; AUC, area under the ROC curve; Acc, accuracy; Se, sensitivity; Sp, specificity; PPV, positive predicted value; NPV, negative predicted value.

## Data Availability

The data that support the findings of this study are available on request from the corresponding author.
